# Heterogeneity in clone dynamics within and adjacent to intestinal tumours identified by Dre-mediated lineage tracing

**DOI:** 10.1242/dmm.046706

**Published:** 2021-01-15

**Authors:** Ann-Sofie Thorsen, Doran Khamis, Richard Kemp, Mathilde Colombé, Filipe C. Lourenço, Edward Morrissey, Douglas Winton

**Affiliations:** 1 Cancer Research UK Cambridge Institute, University of Cambridge, Li Ka Shing Centre, Robinson Way, Cambridge CB2 0RE, UK; 2 University of Oxford, Center for Computational Biology, Weatherall Institute of Molecular Medicine, University of Oxford, John Radcliffe Hospital, Oxford OX3 9DS, UK

**Keywords:** Lineage tracing, Cancer, Intestine, Epithelial

## Abstract

Somatic models of tissue pathology commonly use induction of gene-specific mutations in mice mediated by spatiotemporal regulation of Cre recombinase. Subsequent investigation of the onset and development of disease can be limited by the inability to track changing cellular behaviours over time. Here, a lineage-tracing approach based on ligand-dependent activation of Dre recombinase that can be employed independently of Cre is described. The clonal biology of the intestinal epithelium following Cre-mediated stabilisation of β-catenin reveals that, within tumours, many new clones rapidly become extinct. Surviving clones show accelerated population of tumour glands compared to normal intestinal crypts but in a non-uniform manner, indicating that intra-tumour glands follow heterogeneous dynamics. In tumour-adjacent epithelia, clone sizes are smaller than in the background epithelia, as a whole. This suggests a zone of ∼seven crypt diameters within which clone expansion is inhibited by tumours and that may facilitate their growth.

## Abstract (precis)

**Summary:** A novel Dre^Pr^ recombinase used for sequential lineage tracing in mouse intestinal tissue identifies clonal heterogeneity within tumours, and impaired clonal dynamics in adjacent epithelia may promote tumour growth.

## INTRODUCTION

Mouse models of pathology in which phenotypes are somatically induced by the directed expression of recombinases have become a ubiquitous tool across all branches of the medical sciences. Currently there are over 4000 mouse lines engineered for this purpose [Friedel et al., 2007; The Jackson Laboratory (http://www.informatics.jax.org)]. Activation of recombination in adult tissues is highly efficient and can result in altered cellular behaviours that can change over time due to adaptation, cellular exhaustion or progression, and consequently display different phenotypes that may reflect different disease settings. Examples include arteriosclerosis (Ishibashi et al., 1993), diabetes (Zhang et al., 1994), inflammation (Stremmel et al., 2017), Alzheimer's disease (Matsuda et al., 2008), Parkinson's disease (Choi et al., 2017) and cancer (Cheung et al., 2009).

An important aspect of the phenotypic characterisation of affected tissues following recombination often includes lineage tracing, in which the origins and fate of individual cells and their descendants are followed over time using acquired expression of a cell-autonomous reporter gene. Problematic in such lineage-tracing experiments is that reporter expression is often dependent on the activity of the same recombinases acting to induce the phenotype of interest. This is a major limitation as the level and timing of recombination required for lineage tracing may be very different from that needed to induce the phenotype, although re-switchable cassettes can sometimes be employed (Schepers et al., 2012).

The most widely used DNA recombinases in *in vivo* mouse models are Cre and Flp (Sadowski, 1995; Sternberg and Hamilton, 1981). Historically, most cancer-associated conditional alleles contain pairs of loxP sites for Cre-driven recombination: e.g. *Tp53*^flox2-10^ (Marino et al., 2000), *Apc*^flox^ (Cheung et al., 2009) and *Ctnnb1*^flox(ex3)^ (Harada et al., 1999). Some frt alleles for Flp-driven recombination are also available: e.g. *Kras^fsfG12D^* (Young et al., 2011). To employ recombinases sequentially requires independent spatiotemporal control of the activity of different DNA-recombinases, which can only occur if they are expressed under different promoters and/or activated by different ligands.

The novel Dre DNA recombinase, discovered in a screen for Cre-like enzymes (Sauer and McDermott, 2004), recognises 32 bp rox sites. The Dre/rox system does not cross-react with the Cre/loxP system (Anastassiadis et al., 2009). Dre has been used in conjunction with Cre and Flp to identify cell populations defined by differential promoter activity (Hermann et al., 2014; Madisen et al., 2015; Plummer et al., 2015; Sajgo et al., 2014). An inducible and functional Dre^Pr^ fusion protein (Dre fused to the human progesterone receptor) that can be activated by the synthetic analogue Ru486 has been described (Anastassiadis et al., 2009) but has been used *in vivo* only in a single zebrafish study (Park and Leach, 2013). Here, we employ Dre^Pr^ for somatic studies in adult mice and demonstrate that it can be used in combination with tamoxifen-inducible Cre^Ert^ alleles to initiate lineage tracing at any time following the activation of a Cre-mediated phenotype. The method is applied to determine the altered fate and clone dynamics of stem cell populations within and adjacent to intestinal tumours induced by stabilisation of β-catenin.

## RESULTS

### RDre^Pr^ mice have widespread Dre expression

The RDre and RDre^Pr^ animals were created by homologous recombination of targeting vectors into the *R26* locus in mouse embryonic stem cells as described previously (Vooijs et al., 2001) (Fig. S1A). A rox-STOP-rox (rsr) tdTomato (tdTom) reporter line (*R26*^rsrtdTom^) was generated by germline deletion of the loxP-STOP-loxP (lsl) cassette from the *R26*^rsrlslTdTom^ allele (The Jackson Laboratory, stock number 021876). To investigate Dre activity in different tissues, RDre and RDre^Pr^ animals were crossed to these *R26^rsrtdTom^* reporter mice to generate compound *RDre;R26^rsrtdTom^* and *RDre^Pr^;R26^rsrtdTom^*, referred to here as RDre;RtdTom^rsr^ and RDre^Pr^;RtdTom^rsr^, respectively.

First, adult RDre;RtdTom^rsr^ mice were analysed and the widespread activity of Dre across many tissues was confirmed by immunohistochemistry (IHC) for tdTom expression (Fig S1B, Fig. S2). However, cells in the outermost layer of the epidermis and bone cartilage did not express any tdTom (Fig S1B, Fig. S2). Next, to investigate Dre^Pr^ activity after Ru486 exposure, RDre^Pr^;RtdTom^rsr^ animals had 1, 2 or 3, 90-day slow release pellets containing 10 mg/pellet Ru486 implanted subcutaneously. At 75 days post implantation, animals were sacrificed, and tissues from different germ layers were collected for tdTom expression analysis. Importantly, the Dre^Pr^ fusion mice showed a complete absence of tdTom expression in all tissues in uninduced mice (Fig S1B, Fig. S2). In contrast, following induction with Ru486, sporadic tdTom expression was observed in many tissues. Epithelial cells and/or clones expressing tdTom were observed in endoderm-derived intestine, stomach, liver, pancreas, mesoderm-derived spleen and kidney, and ectoderm-derived skin (Fig. S1B). Furthermore, quantitative flow cytometry confirmed that intestinal epithelial cells were activated by Ru486 pellets in a dose-responsive manner (Fig. S3A-C). Of note, not all tissue types analysed expressed tdTom after Ru486 exposure, including lung, heart, bone and tongue (Fig. S2). The observation that these tissues did show widespread recombination in RDre;RtdTom^rsr^ mice suggests transcriptional silencing at the *ROSA* locus in these tissues in adult mice, or that Ru486 does not reach all tissues, as described previously for Cre recombinase and tamoxifen (Sinha and Lowell, 2017; Vooijs et al., 2001). These results show that Dre^Pr^ has no background activity and is activated by Ru486 in various tissues, including the intestinal epithelium.

### Dre^Pr^ can be clonally induced in epithelial cells

**Fig. 1.**
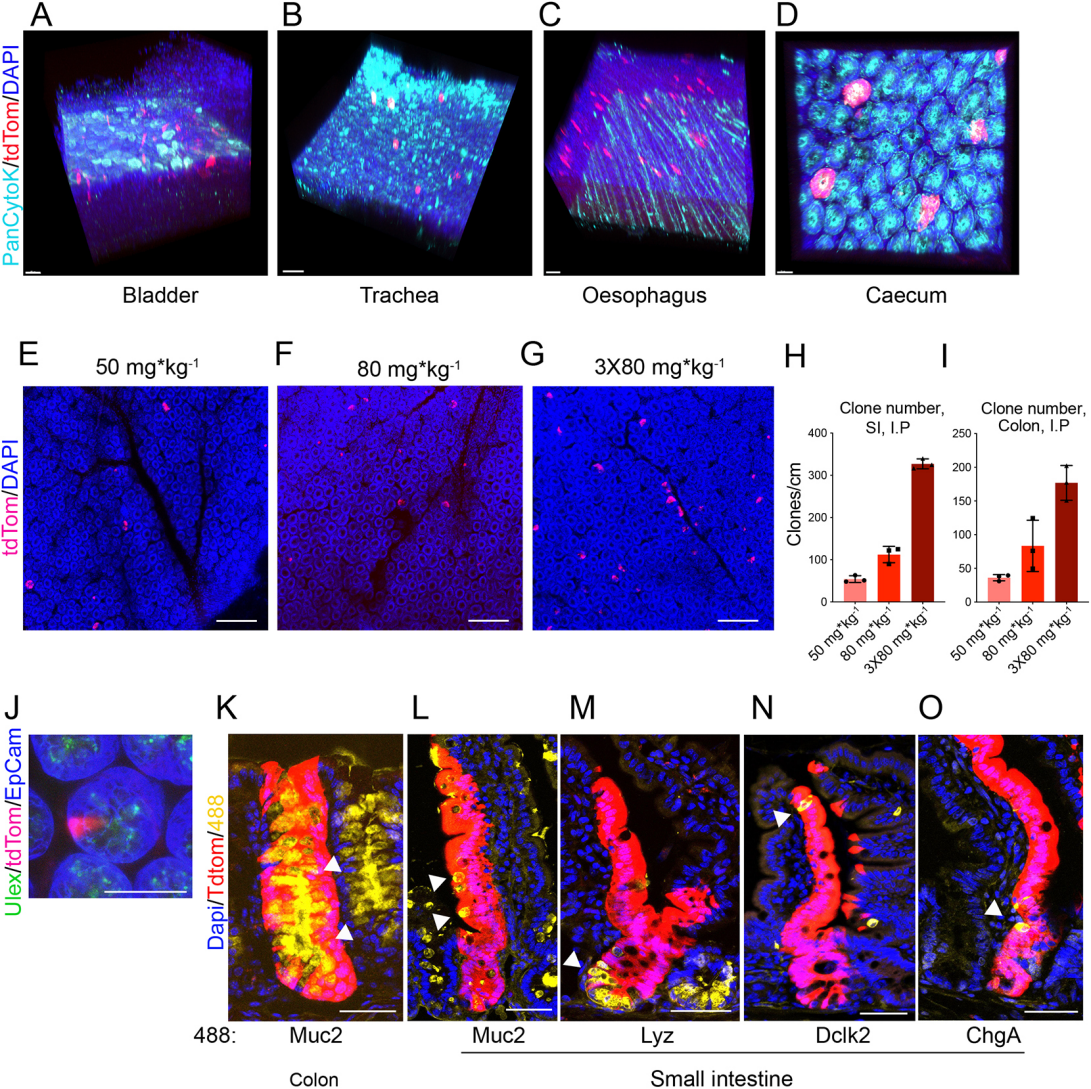
**Clonal recombination induced by Dre^Pr^.** (A-D). Expression of tdTomato in tissues from RDre^Pr^;RtdTom^rsr^ animals imaged 14 days following i.p. injection of 240 mg Ru486. (E-G) Confocal images showing expression of tdTomato in the crypts of small intestinal whole mounts of RDre^Pr^;RtdTom^rsr^ animals 14 days following i.p. injection of Ru486 at the doses shown. (H,I) Bar graph showing quantification of the number of tdTom^+^ crypt clones/cm in small intestinal (H) or colon tissue (I), (*n*=3; data are mean±s.d.). (J) Small intestinal crypt from a RDre^Pr^;RtdTom^rsr^ animal, 24 h after receiving 50 mg/kg Ru486 by i.p. injection, showing a single tdTomato^+^ cell not expressing a Paneth cell marker (*Ulex* lectin). (K-O) Confocal images of tdTomato^+^ clones (arrowheads) from the same animals as for E-I. Colon section visualised for goblet cells (Muc2) (K). Small intestinal sections (L-O) visualised for goblet cells (Muc2) (L), Paneth cells (lysozyme) (M), tuft cells (Dlck2) (N) and enteroendocrine cells (chromogranin A) (O). Scale bars: 50 µm.

Next, the ability of Dre^Pr^ to operate as a lineage-tracing tool in epithelial tissues was investigated. Lineage tracing is commonly carried out by pulse chase experiments (Ghosh et al., 2011; Giroux et al., 2017; Van Keymeulen et al., 2011; Papafotiou et al., 2016; Snippert et al., 2010; Vermeulen et al., 2013). To investigate the activity of Dre^Pr^ after a pulse of inducer, Ru486 was administered to RDre^Pr^;RtdTom^rsr^ mice by intraperitoneal (i.p.) injection at doses of 50 or 80 mg/kg (single injections), or 240 mg/kg (80mg/kg administered on three consecutive days). After 14 days, animals were culled and the bladder, trachea, oesophagus and caecum were analysed by fluorescent microscopy (Fig. 1A-D). This revealed that tdTom^+^ clones could be observed in all tissues (Fig. 1A-D). Additionally, clones were also observed in the intestine (Fig. 1E-G) and the number of clones observed in the small intestine and colon 14 days post induction increased in a dose-responsive fashion in both (Fig. 1H,I). The number of clones/cm in the small intestine was ∼100 versus ∼300 following a single or triple dose of 80 mg/kg Ru486, respectively, and indicated a near linear accumulation of signal (Fig. 1H,I). Twenty-four hours after Ru486 administration, single tdTom^+^ cells could be observed in the bottom of intestinal crypts (Fig. 1J). Over time, tdTom could be observed in whole crypts and villi after Dre^Pr^ induction, both in the small intestine and colon (Fig. 1K-O). These clones contained goblet, Paneth, Tuft and enteroendocrine cells (Fig. 1K-O), underlining that Dre^Pr^ was activated in single clonal intestinal stem cells, and that these cells can give rise to differentiated daughter cells.

For clonal lineage-tracing experiments to be informative, the frequency of clone induction has to be low enough to avoid clonal collisions over the time course of the experiment but high enough to permit quantification within a defined region of the tissue. Here, we found that 50 mg/kg Ru486 was the optimal pulsing dose in lineage-trace experiments, as this dose appeared to hit less than one stem cell per crypt at early time points (Fig. 1J), and induced an appropriate sparsity of clones at later time points (Fig. 1E,H,I).

### Lineage tracing and quantitative inference of intestinal stem cell dynamics using Dre^Pr^

**Fig. 2.**
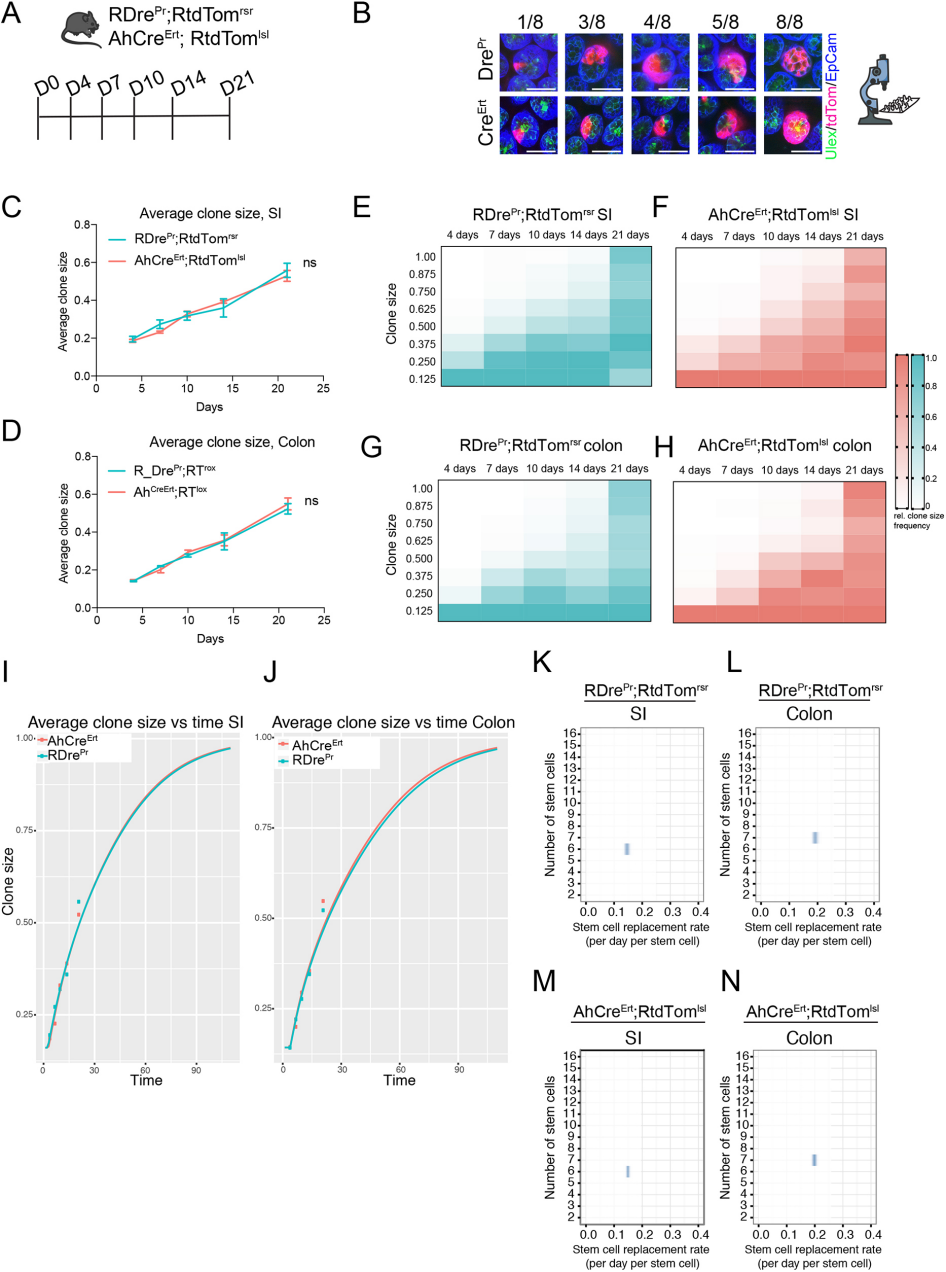
**Stem cell dynamics inferred from intestinal clone size distributions in RDre^Pr^;RtdTom^rsr^ mice.** (A) Schematic of the experimental set-up. RDre^Pr^;RtdTom^rsr^ and AhCre^Ert^;RtdTom^lsl^ animals were administered a single dose of 50 mg/kg Ru486 or 40 mg/kg β-naphthoflavone plus 0.15 mg tamoxifen, respectively. (B) Confocal microscopy images of small intestinal clones demonstrating segmental scoring in eighths of clone sizes (viewed from crypt base) from RDre^Pr^;RtdTom^rsr^ (Dre^Pr^) and AhCre^Ert^;RtdTom^lsl^ (Cre^Ert^) animals. Scale bars: 50 µm. (C,D) Average clone sizes with time in small intestine (SI) (C) and colon (D) derived from RDre^Pr^;RtdTom^rsr^ and AhCre^Ert^;RtdTom^lsl^ animals (*n*=3; data are mean±s.d.). (E-H) Heatmap representing colour-coded clone size prevalence over time during lineage tracing in RDre^Pr^;RtdTom^rsr^ small intestine (E) and colon (G), or AhCre^Ert^;RtdTom^lsl^ small intestine (F) and colon (H). Darkest colour corresponds to most prevalent clone sizes at a given time point. (I,J) Mathematical modelling (line) showing predicted average clone sizes from day 0-100 in small intestine (I) and colon (J), based on the data shown in C and D (points). (K-N) Inferred stem cell number per crypt (*y*-axis) and stem cell replacement rate per stem cell per day (*x*-axis) in small intestine (K) and colon (L) of RDre^Pr^;RtdTom^rsr^ animals, and in small intestine (M) and colon (N) of AhCre^Ert^;RtdTom^lsl^ animals.

The efficacy of Dre^Pr^ for lineage tracing was investigated in the mouse intestine by performing a direct quantitative comparison of epithelial clone size distributions with those obtained in a previously employed Cre-based model (Kemp, 2004; Vermeulen et al., 2013). Clones were induced in AhCre^Ert^;RtdTom^lsl^ mice by a single induction dose (40 mg/kg β-naphthoflavone and 0.15 mg tamoxifen), and RDre^Pr^;RtdTom^rsr^ mice received a single dose of 50 mg/kg Ru486. Small intestine and colon from both strains were analysed at 4, 7, 10, 14 and 21 days post induction, and fluorescence microscopy of whole-mounted tissues was employed to score the relative sizes of tdTom^+^ clones in intestinal crypts (Fig. 2A,B). The average clone sizes and changes in the clone size distribution with time in the small intestine and colon were found to be remarkably similar in the two models (Fig. 2C-H).

Changes in clone size distributions with time arise from a neutral drift pattern of stem cell renewal that is characterised in each crypt as a one-dimensional random walk on a ring of N stem cells, each with a daily replacement rate *λ* (Kozar et al., 2013; Lopez-Garcia et al., 2010). For RDre^Pr^;RtdTom^rsr^ and AhCre^Ert^;RtdTom^lsl^ animals (Fig. 2I-N), the model predicted average clone sizes over 100 days based on the lineage-tracing data (Fig. 2I,J). The analysis inferred N to be 6 and 7 in the small intestine and colon, respectively, in both the RDre^Pr^;RtdTom^rsr^ and AhCre^Ert^;RtdTom^lsl^ models (Fig. 2K-N). Furthermore, *λ* was estimated to be 0.15 versus 0.15 in the small intestine and 0.19 versus 0.20 in the colon of RDre^Pr^;RtdTom^rsr^ and AhCre^Ert^;RtdTom^lsl^ animals, respectively (Fig. 2K-N). Importantly, the average clone sizes presented here, and the inferred values for *N* and *λ*, were similar to those previously reported using alternative Cre recombination models or orthogonal approaches (Kozar et al., 2013; Vermeulen et al., 2013). Taken together, these observations show that the epithelial behaviours of intestinal stem cells are not perturbed by either Dre^Pr^ expression or the treatment with Ru486, indicating that Dre^Pr^-traced stem cells have a non-biased neutral behaviour.

### Dre^Pr^ can trace single-cell derived clones in intestinal tumours

Performing lineage tracing within overt or developing Cre-mediated pathologies requires a Cre-independent mechanism for reporter activation. To test the ability of Dre^Pr^ to act in this way, a Cre-induced intestinal tumour model based on stabilisation of β-catenin was employed. In *Ctnnb1*^flox(ex3)^ mice, the removal of floxed exon 3 is sufficient to induce development of intestinal tumours (Harada et al., 1999). Here, AhCre^Ert^ was used to induce *Ctnnb1* recombination and tumour initiation, and Dre^Pr^ was used to trace clones derived from single cells within nascent and established tumours.

**Fig. 3.**
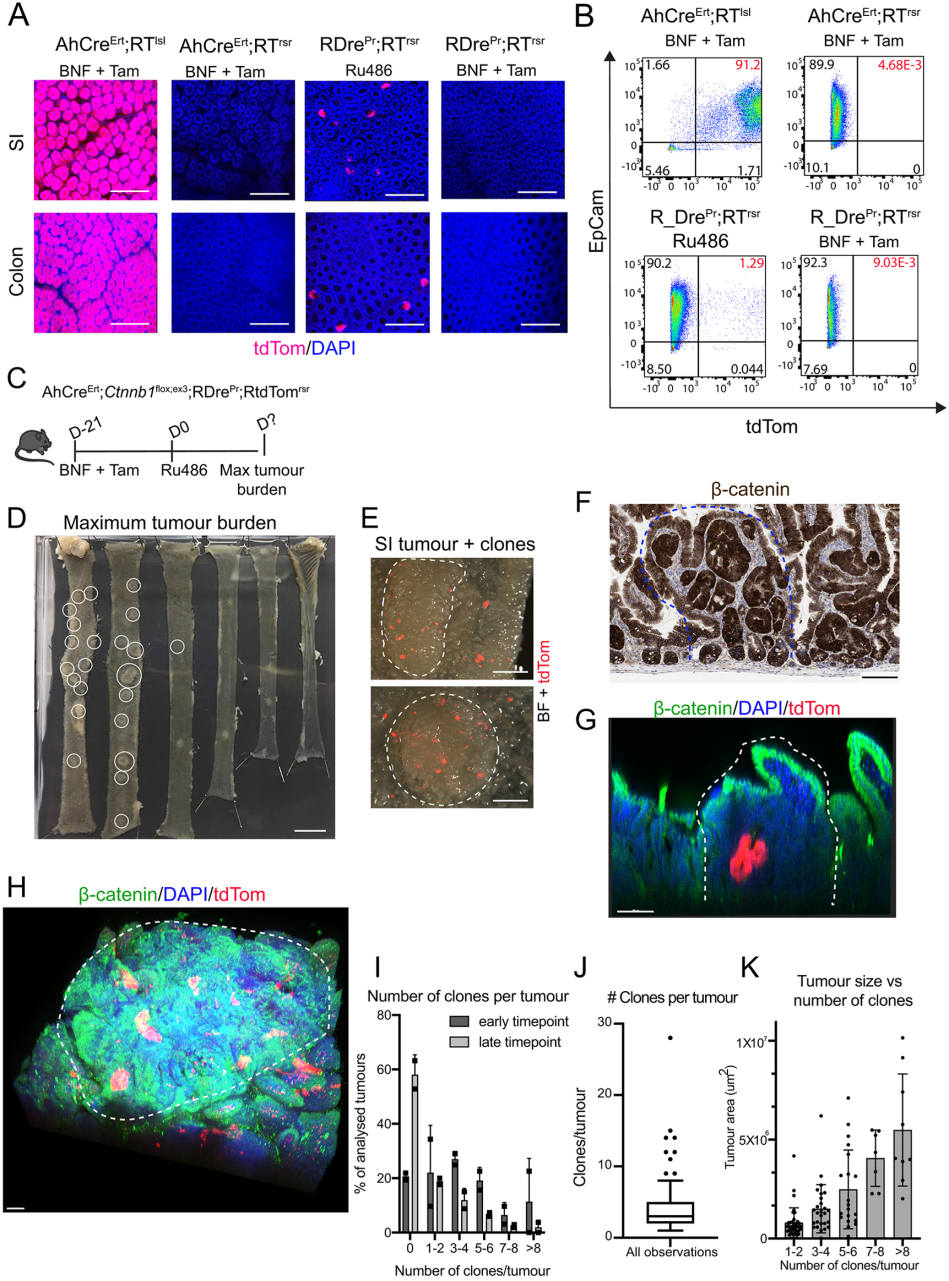
**Activation of Dre^Pr^ allows lineage tracing within intestinal tumours initiated by Cre-mediated stabilisation of** β**-catenin.** (A,B) β-naphthoflavone (BNF) and tamoxifen (Tam) induces recombination of loxP but not rox sites in AhCre^Ert^ animals, and not at rox sites in RDre^Pr^ animals. (A) Confocal microscopy images of small intestine and colon from AhCre^Ert^;RtdTom^lsl^, AhCre^Ert^;RtdTom^rsr^ and RDre^Pr^;RtdTom^rsr^ animals treated as shown. (B) Flow cytometry quantification of tdTom^+^ cells in proximal small intestine (SI) from the above animals. Cells were stained with EpCam (Alexa Fluor 647) and DAPI. The numbers in the quadrants indicate population percentages. The percentages in the left and right quadrants indicate absence or induction of tdTom, respectively. The percentages in the upper right quadrant (in red) indicate the proportion of EpCam^+^/tdTom^+^ cells. (C) Schematic representation of experimental set-up. Tissue was collected at the humane endpoint. (D) Whole mount of intestine at maximum tumour burden. Macroscopic tumours are circled. (E) Dissection microscopy picture of whole-mounted small intestinal tumour (white-dashed line) with tdTom^+^ clones. Overlay of the brightfield (BF) and 555 nm channel (tdTom). (F) Section showing intestinal tumour (dashed blue line) with IHC for β-catenin. (G) Cross-section of a tumour (dashed white line), containing a tdTom^+^ clone. (H) Representative confocal microscopy image of multiple tdTom^+^ clones in tumours stained for β-catenin (dashed white line). (I) Bar graph summarising the distribution of the number of tdTom^+^ clones (0 to more than 8) in tumours from mice culled 13 days (early) or 17-19 days (late) post Ru486 induction (*n*=62 and 121, respectively). Each data point shows mean number of tumours per mouse. (J) Boxplot displaying the average number of clones per tumour (of all tumours containing clones) (*n*=100). Data outside the 10th and 90th percentile is displayed as single data points. (K) Bar graph showing the number of tdTom^+^ clones (1 to more than 8, binned by 2) in all tumours based on tumour area (µm^2^). Each data point is a tumour (*n*=100; data are mean±s.d.). Scale bars: 200 µm (A); 1 cm (D); 1 mm (E); 100 µm (F-H).

To determine the number of traceable cells per tumour, *Ctnnb1* was first recombined in the intestinal epithelium of AhCre^Ert^;*Ctnnb1*^lox(ex3)^;RDre^Pr^;RtdTom^rsr^ animals by activating AhCre^Ert^ with β-naphthoflavone and tamoxifen. Importantly, these drugs did not induce tdTom expression (Fig. 3A,B; Fig. S4). At 21 days post *Ctnnb1* recombination, lineage tracing in tumours was initiated by RDre^Pr^ induction and mice were then aged until maximum tumour burden, 13 days (*n*=2), 17 days (*n*=1) and 19 days (*n*=1) days post Ru486 induction (Fig. 3C). This protocol produced intestinal tumours expressing stabilised β-catenin that contained tdTom^+^ clones (Fig. 3D-G).

Each whole-mounted tumour was subjected to tissue clearing and fluorescence microscopy to score the number of clones per tumour (Fig. 3H-J). Out of 184 tumours analysed, 84 did not contain clones (45%). Dividing the data into early [day 13 post Ru486 (*n*=2)] and late [day 17 and 19 post Ru486 (*n*=2)], demonstrated that at the earlier time tumours contained more clones than later ones; 20% and 60% of tumours containing no clones, respectively (Fig. 3I). Moreover, there appeared to be a trend that early tumours with clones contained more clones than late tumours (Fig. 3I). The average number of clones per tumour (of all tumours with clones, *n*=100) was found to be 4.4 and the highest number of clones in one tumour was 28 (Fig. 3J). Tumour size appeared to correlate with number of clones, so that larger tumours contained a higher number of clones than smaller tumours (Fig. 3K). Together, these results suggest that tumour size dictates the number of clones per tumour that can be induced by Dre^Pr^, probably reflective of a larger target population present at the time of induction. In addition they demonstrate that, as predicted by a neutral competition model of stem cell replacement, there are clonal extinction events occurring within developing tumours with time.

**Fig. 4.**
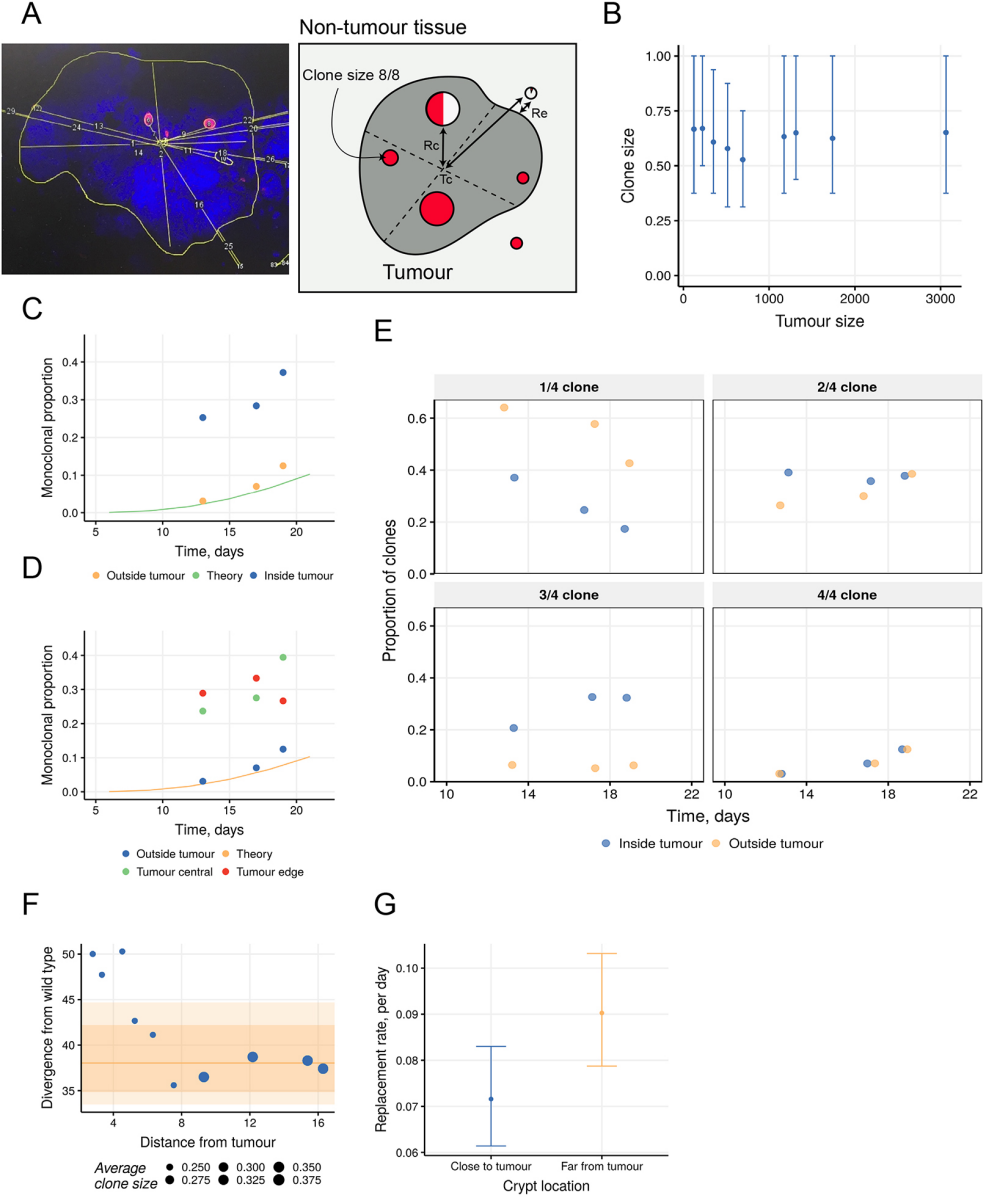
**Stem cell dynamics are accelerated within *Ctnnb1*-driven tumours but inhibited adjacently to them.** (A) Schematic diagram and real example of measurements used to characterise clones inside and outside of tumours. (B) Graph displaying average clone size across binned median of tumour sizes. Each bin contains 50 clones. Points and bars show median and interquartile range. (C) Graph comparing the proportion of surviving clones that have monoclonally converged within tumours and in surrounding tissue. (D) As in C but with the intra-tumour data split into those clones that are further from (tumour edge) or closer to the Tc (falling without or within 75% of the average tumour radius, respectively). (E) The proportion of surviving non-fixed clones (in crypt quartiles) inside and outside tumours evolve differently in time when the monoclonal proportion inside tumours has been rescaled to be identical to the monoclonal proportion outside tumours. (F) Divergence from theoretical wild type of the clonal dynamics in crypts occupying adjacent intestinal epithelia surrounding β-catenin tumours. Clones are grouped into overlapping bins containing equal numbers of crypts (100 clones per bin) increasing in distance from the edge of the tumour. Line and ribbon shading show the 80% and 95% credible intervals around the median of 1000 simulated datasets of 100 wild-type crypts. Point sizes show the average clone size in each bin. Distances are measured in crypt diameters. (G) The daily replacement rate *λ* of stem cells undergoing neutral drift is inferred for clones in tumour-adjacent epithelia. Close to tumour (Re<7 crypt diameters), *λ*=0.07 [80% confidence interval (c.i.): 0.061-0.083]; far from tumour (Re≥7 crypt diameters), *λ*=0.09 (80% c.i.: 0.079-0.103).

Next, we sought to exploit the different times that mice were culled post lineage induction to derive the neutral behaviours of clones in tumour glands, and to compare these to that of normal epithelia and of the background tumour-prone epithelium. Clone sizes were quantified by classifying each clone by the proportion of the gland it occupied (as a fraction of 8) within and outside tumours, and their spatial distribution determined with respect to the tumour centre (Tc) (Fig. 4A) (see Materials and Methods). Analysis within all tumours showed no clear relationship between clone size and tumour size (Fig. 4B). However, although changes in tumour clone size distributions between 13 and 19 days were approximately parallel to that predicted for wild-type tissue and the background tissue in the same animals, there was a massive over-representation of monoclonal glands within tumours that did not fit with the trajectory of these later time points (Fig. 4C). Specifically, at 13, 17 and 19 days post induction, 25.3% (69 of 273), 28.4% (23 of 81) and 37.2% (32 of 86) of surviving clones inside tumours were comprised of monoclonal glands, whereas outside of tumours the corresponding values were 3.1% (4 of 130), 7.0% (5 of 71) and 12.5% (8 of 64). Wild-type neutral drift theory in the small intestine predicts 2.2%, 5.5% and 7.8% at these three time points (Fig. 4C). Together, this suggests that either the clone dynamics of tumours changes over time or that they are heterogeneous with some glands showing accelerated dynamics. The spatial distribution of clones and tumour size were explored to determine whether different behaviours related to proximity to the tumour edge or the size of the tumour; however, no obvious trend was observed (Fig. 4D; Fig. S5A). To explore whether this effect was due to some glands showing accelerated dynamics (i.e. the monoclonal glands), the monoclonal proportion in the tumour dataset was rescaled to be identical to the data recorded outside of tumours. This analysis showed that even after rescaling, the intracrypt clonal dynamics within tumours was accelerated compared with that of external crypts (Fig. 4E). Such variable behaviour precludes deriving stem cell metrics using neutral drift theory but indicate that the tumour glands arising from stabilisation of β-catenin all show accelerated but variable clone dynamics leading to monoclonality.

In considering the clone dynamics of the background clones in tumour-bearing mice, we next considered the impact of tumours on their behaviour. The size distribution of clones was determined with respect to their proximity to tumours by applying a rolling window moving away from the tumour that always contained the same number of clones. This analysis revealed that crypts closer to tumours had appreciably smaller clones, reflecting slower drift dynamics and longer times to achieve monoclonality despite not being appreciably different in size (Fig 4F; Fig. S5B). This trend did not vary with the size of tumours (Fig. S5C). Calculation of stem cell replacement rates confirmed that these were reduced in tumour-adjacent crypts (Fig 4G; Fig. S5D). These findings suggest that tumours create an inhibitory local environment that slows stem cell replacement processes in adjacent crypts. Together, these observations show that RDre^Pr^ can be used for lineage tracing to reveal the altered stem cell behaviours arising as a consequence of stabilisation of β-catenin, and in the resultant tumours.

## DISCUSSION

The bulk of somatically induced genetically engineered mouse models depend on the regulated activity of Cre-recombinase. The use of appropriate promoter elements and/or post-translational ligand-dependent regulation allows gene-specific changes to be mediated at different developmental stages, in specified tissues, at known times and to varying extents. In parallel, Cre-activated reporters have offered a route to determining altered cell fates and properties. Here, we have shown that Dre^Pr^ is activated in a dose-dependent manner in various tissues, and can be used for lineage tracing in the small intestine and colon with a robustness that allows for detailed interpretation of quantitative data. In addition, Dre^Pr^ can be combined with Cre-mediated models, as shown by the lineage tracing following stabilisation of β-catenin driven tumours.

A prerequisite for lineage tracing is to have minimal reporter background recombination in the absence of an inducer. Importantly, RDre^Pr^ mice show no background activity. In contrast, models that rely on highly efficient recombination to achieve tissue-wide alterations in gene expression (e.g. *Villin*^CreErt^ in intestinal studies) are often subject to significant background recombination. It follows that such models are inappropriate for sporadic induced recombination or clonal lineage tracing. Hence, the RDre^Pr^ mouse line is an appropriate tool for low frequency recombination with minimal background.

This is the first description of a recombination driven sequential model for lineage tracing in intestinal tumours. The results show that clone bearing tumours in the AhCre^Ert^;*Ctnnb1*^lox(ex3)^;RDre^Pr^;RtdTom^rsr^ model contain an average of four clones at the end of the experimental animal life span. The surviving clones observed in the 2 to 3 week interval following lineage tracing have predominantly populated whole tumour glands. In this regard, the current findings are consistent with our previous study using an approach of ‘continuous labelling’, in which analysis of spontaneous mutations within the glands of spontaneous *Apc*^min^ adenomas indicated that these are maintained by a small number of clonogenic stem cells between which there is a high rate of replacement. However, here, using the additional resolution of a pulse-chase approach mediated by Dre^Pr^, we further identify heterogeneity in the clone dynamics of individual tumour glands in the *Ctnnb1^l^*^ox(ex3)^ model.

When considering the clonal dynamics of the background epithelium in intestines heavily ‘peppered*’* with tumours, we determined that proximity to a tumour slows the inferred rate of stem cell replacement. Many reports have indicated that the impact of the mutations that cause colorectal cancers by hyperactivating the Wnt signalling pathway also cause induction of secreted negative feedback inhibitors, or of other pathways that would normally act to downregulate Wnt signalling (González-Sancho et al., 2005; Kakugawa et al., 2015; Koo et al., 2012; Mishra et al., 2019). It appears likely that some of these are acting in a non-cell (or gland)-autonomous manner and are additionally slowing the stem cell competitive replacement process in nearby crypts. Determining the significance of this phenomenon is beyond the scope of this report but we speculate that there may be a form of inter-gland competition, such that tumour glands promote their growth by reducing the fitness of adjacent wild-type crypts.

Together, these observations demonstrate the use of mammalian-expressed Dre^Pr^ specifically and of secondary lineage tracing in general to describe and understand complex pathologies that progress with time. The approach may be particularly relevant to documenting the nature and efficacy of therapeutic interventions applied at different stages of disease progression.

## MATERIALS AND METHODS

### Treatment of animals

The mice were housed under controlled conditions (temperature, 21±2°C; humidity, 55±10%; 12 h light/dark cycle) in a specific-pathogen-free (SPF) facility (tested according to the recommendations for health monitoring by the Federation of European Laboratory Animal Science Associations). The animals had unrestricted access to food and water, were not involved in any previous procedures and were test naive. Mice used in this study were 8- to 16-week old males and females of C57BL/6 background. Ru486 (Sigma-Aldrich, M8046) was dissolved in 100% ethyl alcohol (Honeywell, E7023) to make a 50 mg/mL solution, and further diluted into a working solution of 10 mg/mL in 40% cyclodextrin/H_2_O. β-naphthoflavone (Sigma-Aldrich, N3633) was dissolved to a working solution of 8 mg/mL in corn oil (Sigma-Aldrich, C8267). Tamoxifen (Sigma-Aldrich, T5648) was dissolved in 100% ethyl alcohol (Honeywell, E7023) to make a 200 mg/mL solution, and further diluted to a working solution of 20 mg/mL in sunflower oil (Sigma-Aldrich, S5007).

The subcutaneous insertion of Ru486 slow release pellet(s) in mice was carried out by the Biological Resource Unit Core at Cancer Research-UK Cambridge Institute (CRUK CI). Mice receiving pellet insertion surgery were all 8 to10 weeks of age. Mice were placed under general anaesthesia and a 1 cm subcutaneous incision was made on the back flank of each mouse in which one to three Ru486 pellets (Innovative Research of America, 10 mg per pellet, 90 days release, NX-999) were placed. The wound was closed with surgical glue.

### Model creation

The RtdTom^rsrlsl^ mouse was bought from the Jackson Laboratories (stock number 021876). The deletion of the lsl cassette to generate the RtdTom^rsr^ strain was carried out by the CRUK CI Genome Editing Core. RtdTom^rsrlsl^ early embryos were cultured with the cell-permeable TAT-Cre *in vitro*, and embryo transfer was performed as described by Ryder et al. (2014). PCR screening was used to ensure lsl cassette deletion. RDre and RDre^Pr^ were generated in house at the CRUK CI Genome Editing Core in the Biological Resource Unit by embryonic stem cell electroporation and homologous recombination of *R26* targeting vectors, and subsequent oocyte injections. A splice acceptor site was inserted immediately before the Dre sequence and Pr (consisting of the Ru486 responsive mutant hormone binding domain of the human progesterone receptor hPR891; Kellendonk et al., 1996) was fused to the *N*-terminus of Dre (fusion site identical to Anastassiadis et al., 2009) followed by a bovine growth hormone (BGH) polyadenylation signal and inserted into a R26 targeting vector (Vooijs et al., 2001). The RDre model lacks the Pr sequence. Embryonic stem cell (ESC) screening for correct 5′ integration was carried out by PCR amplification with P1 (5′-AGAGTCCTG-ACCCAGGGAAGACATT-3′) and P2 primers (5′-CATCAAGGAAACCCTGGACTACTGCG-3′). 3′ end integration was confirmed with primers P3 (5′-GTCACCGAGCTGCAA-GAACTCT-3′) and R2 (5′-GGTGGTGGTGGTGGCATATACATT-3′). Single copy insertion of the vectors was confirmed by copy number assay before oocyte injection of ESCs.

### Animal genotyping

Genotyping was carried out by Transnetyx Inc.

### Epithelial cell isolation

Small intestine and colon were dissected, flushed with PBS, everted and fed onto a glass rod spiral. They were incubated at 37°C in Hank's Balanced Salt Solution without Ca^+2^ and Mg^+2^, containing 10 µM EDTA and 10 mM NaOH. Epithelial cell release was facilitated using a vibrating stirrer (Chemap). Samples were incubated for 1 h and pulsed every 10 min. Fractions were collected after each pulse, and fresh solution added. Fractions were pooled and washed in cold 2% FBS/PBS. Samples were snap frozen before DNA, RNA or protein isolation, or stained with antibodies for flow cytometry analysis.

### Flow cytometry

Single-cell suspension obtained by trypsin treatment was washed and incubated with an anti-mouse CD326 (EpCAM) Alexa Fluor 647 antibody (1:2000, clone G8.8, BioLegend). DAPI (10 μg/mL) was added to distinguish between live and dead cells. Flow sorting was carried out using a BD FACS Aria SORP (BD Biosciences), using appropriate single-stained and unstained controls. Data analysis was carried out using FlowJo Software.

### Whole mounting

Tissue was cut open, pinned out luminal side up, and fixed for 3 h at room temperature in ice-cold 4% paraformaldehyde (PFA) in PBS (pH 7.4). Whole mounts were washed with PBS and incubated with demucifying solution [3 mg/mL dithiothreitol (DTT), 20% ethanol, 10% glycerol, 0.6% NaCl, and 10 mM Tris, pH 8.2] for 20 min. Mucus was then removed by washing with PBS.

### Lineage tracing and clone quantification

For lineage tracing, mice were induced with a pulse of β-naphthoflavone and tamoxifen (40 mg/kg and 0.15 mg, respectively) or Ru486 (50 mg/kg), and tissue was whole mounted at days 4, 7, 10, 14 and 21 after pulse administration. Clone sizes were scored manually under a fluorescent microscope using the 550 nm filter. Tissue (2 cm) was mounted muscle side up on a glass-slide, and clone sizes were scored as fractions of eight. All tissue was scored blinded. Three mice per time point were quantified. In RDre^Pr^;RtdTom^rsr^ animals, an average of 100-250 and 85-170 clones/animal/time point were quantified in the small intestine and colon, respectively. In AhCre^Ert^;RT^lsl^ animals, an average of 200-300 and 175-300 clones/animal/time point were quantified in the small intestine and colon, respectively. The highest clone numbers were found at the earliest time points. For number of clones/cm quantification, the number of clones was scored in 2-3 cm of whole-mounted tissue, as described above.

### Tissue clearing of tumour tissue

Whole-mounted tumour tissue was fixed in 4% PFA overnight at 4°C. Thereafter, tissue was washed in PBS for 8 h at room temperature. Tissue was then incubated in CUBIC-1A solution [10% Triton X-100, 5% NNNN-tetrakis (2-HP) ethylenediamine (Sigma-Aldrich, 122262), 10% Urea and 25 mM NaCl] with DAPI 1:100 (10 mg/mL stock) at 37°C on a rotator at 60 rpm for a total of 5 days. On days 2 and 4, CUBIC-1A+DAPI was refreshed (Susaki and Ueda, 2016). After CUBIC-1A incubation, tissue was washed in PBS for 2 h and then placed in RapiClear (SunJin Lab, RC152002) and incubated at room temperature until the tissue was see-through. Thereafter, tissue was mounted on 1 mm inserts (iSpacer, SunJin Lab) on glass-slides in RapiClear and imaged using a TCS SP5 confocal microscope (Leica). Image analysis was carried out using iMaris software.

### Antibody staining of whole organs

For whole-mounted intestinal tissue, sections were washed in 0.1% PBS with Tween 20 (PBS-T) for 2 days, blocked in 10% donkey serum in PBS overnight at 4°C and incubated with an anti-mouse CD326 (EpCAM) Alexa Fluor 647 antibody (1:100, clone G8.8, BioLegend 118201), Ulex-Lectin 488 (1:100, Sigma-Aldrich, 19337) and DAPI (10 μg/mL). Finally, the tissue was washed with PBS-T for 1 day before imaging. Whole-mounted intestinal tissue carrying tumours was covered in optimal cutting temperature compound, placed at −80°C overnight, then washed in 0.5% PBS-T for 2 days at 4°C, and blocked in 10% donkey serum overnight. The tissue was then stained with β-catenin (1:100, Cell Signaling Technology, 9587) and DAPI (10 μg/mL in PBS-T for 3 days at 4°C, washed for 1 day, incubated with donkey anti-rabbit 488 secondary antibody (1:500, Thermo Fisher Scientific, A-21206) for 2 days at 4°C, followed by a 1-day wash in PBS-T. Thereafter, the tissue was placed in RapiClear (SunJin Lab, RC152002) and incubated at room temperature for 2 days before imaging. The bladder, trachea, oesophagus and caecum were whole mounted and then incubated in CUBIC1-A for 5 days (see tissue clearing of tumour tissue), washed in 0.5% PBS-T for 2 days at 4°C, blocked in 10% donkey serum in PBS overnight and then incubated with anti-pan cytokeratin (1:100, Abcam, ab236323) and DAPI (10 μg/mL) for 3 days at 4°C. The tissue was washed and incubated with donkey anti-rabbit 488 secondary antibody (1:500, Thermo Fisher, A-21206) for 2 days at 4°C, followed by another 1-day wash in PBS-T before being placed in RapiClear (SunJin Lab, RC152002) and incubated at room temperature until see-through.

### Immunofluorescence

Tissue was excised and fixed for 48 h in 4% PFA in PBS at 4°C, after which it was transferred to 20% sucrose solution. After cryosectioning, antigen retrieval was accomplished by incubating the slides in 1% SDS for 5 min. Blocking was performed with 10% donkey serum. Following washing, primary antibodies were added and incubated overnight at 4°C. The following primary antibodies were used: rabbit FITC-anti-Lyz (1:400, Dako, F037201), rabbit anti-Muc2 (1:50, Santa Cruz Biotechnology, sc-15334), rabbit anti-ChgA (1:100, Abcam, ab15160) and rabbit anti-Dclk1 antibody (1:1000, Abcam, ab31704). Secondary detection was carried out using Alexa Fluor 488 donkey anti-rabbit secondary antibody (1:500, Thermo Fisher Scientific, A-21206) and DAPI (10 μg/mL). Fluorescent imaging was carried out using a TCS SP5 confocal microscope (Leica). Image analysis was carried out using iMaris software.

### Immunohistochemistry

The small intestine and colon were opened and fixed for 24 h in 4% PFA. The tissue was paraffin embedded and sectioned. RFP and β-catenin immunohistochemistry were carried out using a Bond Max autostainer (Leica), with sodium citrate, pH 6.0 (10 mM) antigen retrieval. Slides were blocked with 3% hydrogen peroxide, followed by incubation using an Avidin/Biotin Blocking Kit (Vector Laboratories). Anti-RFP (1:100, Abcam ab34771) and β-catenin (BD biosciences, 610154, 0.25 μg/mL) primary antibodies were used. For β-catenin IHC, a mouse-on-mouse blocking step was added (Vector Laboratories, MKB-2213). Secondary antibodies used were biotinylated donkey and biotinylated donkey anti-rabbit (1:250, Jackson ImmunoResearch, 711-065-152) and biotinylated rabbit anti-mouse IgG1 (1:500, Abcam, ab125913). Slides were incubated with streptavidin coupled with horseradish peroxidase (HRP), and colour developed using diaminobenzidine (DAB) and DAB Enhancer (Leica).

### Clonal analysis in tumours

To quantify clone sizes in and outside tumours, as well as their spatial distribution, we defined each Tc, distance from clones inside to Tc, distance from clones in adjacent tissue to tumour edge, clone sizes (as a fraction of 8), as well as crypt sizes of clones in each tiled image from animals presented in Fig. 3C-K. In four animals a total of 100 (25 per animal) tumours were analysed. Analysis was carried out using Fiji software.

## Computational analysis

### Neutral drift model

As described previously (Lopez-Garcia et al., 2010; Snippert et al., 2010; Vermeulen et al., 2013; Kozar et al., 2013), the intra-crypt clonal dynamics of stem cells in the murine small intestine and colon can be accurately described via the stochastic neutral drift theory, wherein a subset *N* of equipotent crypt base columnar cells undergo a continuous process of replacing their neighbours or themselves being replaced, with replacements occurring at a daily rate *λ*. The time evolution of this stochastic clonal expansion and contraction, assuming a single stem cell is labelled at time *t*=0, can be captured via solution of the continuous-time master equation for a one-dimensional random walk with absorbing boundaries at 0 and *N* (clonal extinction and monoclonal convergence, respectively):
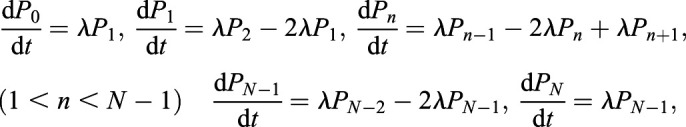
where *P_n_*(*t*) is the probability that a clone has reached a size *n* by time *t*, and *P*_1_(0)=1 and *P_n_*_≠1_(0)=0 define the initial conditions. The solution of the above system (as described by Lopez-Garcia et al., 2010) is:
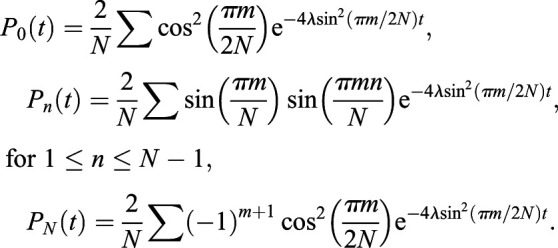


In order to fit the neutral drift model to count data of clone sizes, there are three adjustments that need to be made: (1) the probabilities *P_n_*(*t*) must be rescaled to model surviving clones, *P*′*_n_*=*P_n_*/(1−*P*_0_), such that the *P*′*_n_* sum to one for *n*>0 for all t; (2) the *P*′*_n_* must be redistributed into the number of bins that were used to score the clone sizes, in this case eighths; and (3) the delay between tamoxifen administration and the accrual of the stem cell label is included as a time delay parameter *τ*. Then, count data X is modelled as a multinomial with *P*′*_n_* – now a function of the model parameters *λ*, *N* and *τ* – as the case probabilities:
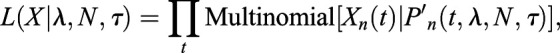
where *X_n_*(*t*) is the vector of counts of clone sizes 1≤*n*≤*N* observed at time *t*. The priors and associated hyperparameters, which were chosen to be uninformative, were:
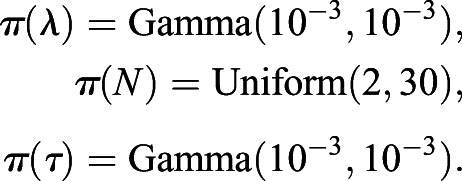


Using this Bayesian inference model, Markov Chain Monte Carlo (MCMC) simulations were used to produce draws of the neutral drift parameters for the wild-type small intestine, as shown in Fig. 2K. In all MCMC sampling herein, 40,000 iterations were performed on two parallel chains each with a burn in of 5000 iterations, with the results thinned by a factor of 20. From this inference, only a well-resolved value for the time delay parameter was desirable to use in the tumour analysis. The time points were 4, 7, 10, 14 and 21 days post induction, at which 692, 595, 592, 457 and 302 clones were observed, respectively. The parameter value was found to be *τ*=2.45 (95% credible interval: 2.07–2.75). This was taken as the Dre technology-intrinsic time delay and *τ*_glob_=*τ* was defined for use in all future computations.

For divergence from wild type, all tumour lineage-tracing data (internal and external tissue) was taken from the proximal small intestine. Therefore, the pulse-chase neutral drift theory, with *N*_WT_=5 stem cells with *λ*_WT_=0.1 daily replacements (Kozar et al., 2013), was used as the baseline wild-type behaviour for investigations examining whether and by how much the observed data diverges from it. The theoretical results were split symmetrically into eight bins to match the eighths used to measure the clone sizes, though symmetry was broken to maintain the identity of monoclonals. This gives a theoretical distribution of clone sizes *P*′*_n_*(*t*), 1≤*n*≤8, occupied by surviving clones at a given time *t* post labelling, as defined in the neutral drift model section.

To investigate the effect on clonal dynamics of proximity to a tumour, the putatively normal tissue surrounding tumours was binned radially from the edge of the tumour outwards, increasing in distance from the tumour (like the rings of a target). To achieve statistical power and allow between-bin comparison, these bins were allowed to overlap so that each bin contained 100 clones (thus each clone was assigned to one or more bins) but the median distance of crypts from the tumour edge in subsequent bins increased monotonically. The likelihood under the theoretical multinomial model for the pooled clone sizes in bin *b* and time point *t*, *C_b_*(*t*) is given by:
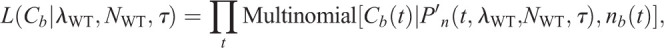
where *n_b_*(*t*) is the number of crypts in bin *b* that arose from time point *t*. The value of the time delay *τ* is fixed at the value inferred from the control data, as described in the previous section. The likelihood for bin *b* explains how well the theoretical wild-type model describes the data observed in that spatial bin in tissue external to a tumour.

In order to interpret these results, a null distribution on the likelihood was created by simulating counts from the theoretical multinomial model, *C*_sim_∼Multinom(*P*′*_n_*(*t*), *n*_b_), where *n*_b_ is the bin size (100 clones) and where the proportion of clones from each time point was the same as that in the data. Counts were generated 1000 times, and the likelihood of each simulation under the theoretical model was calculated. The credible intervals resulting from these simulations were used to judge whether the clones in the binned data were undergoing perturbed dynamics or were within the variability expected from finite sampling.

### Quantifying the clonal dynamics close to tumours

To quantify the effect of tumours on the clonal dynamics in tumour-adjacent crypts, the dataset was split into two subsets: those crypts displaying dynamics not well described by the wild-type theory (those outside the 95% credible interval of the simulated null distribution, 140 crypts) and those displaying dynamics that are well described by the wild-type theory (those inside the 95% credible interval, 125 crypts). The value of the radial distance cutoff that this corresponded to was 6.99 crypt diameters (454 μm). The neutral drift replacement rate *λ* was inferred for each data set using a modified version of the MCMC algorithm described above, wherein the delay parameter and the number of stem cells were fixed to *τ*=*τ*_glob_ and *N*=*N*_WT_ in order to make the replacement rate identifiable.

### Binning clones

Two methods of binning were used to group clones into statistically powered subsets with respect to a measurement, say X: (1) overlapping bins with a constant number of clones in each but a variable width in the parameter X (used in Fig 4F and Fig. S5B,C, with Fig. 4B having an overlap of zero bar the point at the far right, which overlaps its neighbouring point in order to maintain constant bin size); and (2) non-overlapping bins that equally divide the parameter X such that each bin can have a different number of clones (used in Fig. S5A).

### Rescaling monoclonals

To compare the evolution in time of partial clone sizes (those clones that have not become fixed in a gland) within tumours and in tumour-adjacent epithelia, the large monoclonal bias inside tumours was first scaled away (this is necessary as we work in proportions). The rescaled and re-normalised intra-tumour clone sizes 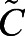 were calculated as:
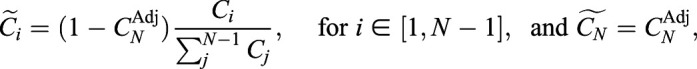
where *C* is the raw intra-tumour clone size distribution and 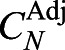 is the proportion of monoclonals in the clone size distribution of the tumour-adjacent tissue. The results are shown in Fig. 4E.

## Supplementary Material

Supplementary information

## References

[DMM046706C1] Anastassiadis K., Fu J., Patsch C., Hu S., Weidlich S., Duerschke K., Buchholz F., Edenhofer F. and Stewart A. F. (2009). Dre recombinase, like Cre, is a highly efficient site-specific recombinase in *E. coli*, mammalian cells and mice. *Dis. Model. Mech.* 2, 508-515. 10.1242/dmm.003087 19692579

[DMM046706C2] Cheung A. F., Carter A. M., Kostova K. K., Woodruff J. F., Crowley D., Bronson R. T., Haigis K. M. and Jacks T. (2009). Complete deletion of Apc results in severe polyposis in mice. *Oncogene* 29, 1857-1864. 10.1038/onc.2009.457 20010873 PMC2990498

[DMM046706C3] Choi W.-S., Kim H.-W., Tronche F., Palmiter R. D., Storm D. R. and Xia Z. (2017). Conditional deletion of Ndufs4 in dopaminergic neurons promotes Parkinson's disease-like non-motor symptoms without loss of dopamine neurons. *Sci. Rep.* 7, 44989 10.1038/srep44989 28327638 PMC5361188

[DMM046706C100] Friedel R. H., Seisenberger C., Kaloff C. and Wurst W. (2007). EUCOMM – the European conditional mouse mutagenesis program. *Brief. Funct. Genomic Proteomic.* 6, 180-185. 10.1093/bfgp/elm022. PMID 17967808. 17967808

[DMM046706C5] Ghosh M., Brechbuhl H. M., Smith R. W., Li B., Hicks D. A., Titchner T., Runkle C. M. and Reynolds S. D. (2011). Context-Dependent Differentiation of Multipotential Keratin 14–Expressing Tracheal Basal Cells. *Am. J. Respir. Cell Mol. Biol.* 45, 403-410. 10.1165/rcmb.2010-0283OC 21131447 PMC3175566

[DMM046706C6] Giroux V., Lento A. A., Islam M., Pitarresi J. R., Kharbanda A., Hamilton K. E., Whelan K. A., Long A., Rhoades B., Tang Q. et al. (2017). Long-lived keratin 15+ esophageal progenitor cells contribute to homeostasis and regeneration. *J. Clin. Invest.* 127, 2378-2391. 10.1172/JCI88941 28481227 PMC5451220

[DMM046706C7] González-Sancho J. M., Aguilera O., García J. M., Pendás-Franco N., Peña C., Cal S., de Herreros A. G., Bonilla F. and Muñoz A. (2005). The Wnt antagonist DICKKOPF-1 gene is a downstream target of β-catenin/TCF and is downregulated in human colon cancer. *Oncogene* 24, 1098-1103. 10.1038/sj.onc.1208303 15592505

[DMM046706C8] Harada N., Tamai Y., Ishikawa T.-O., Sauer B., Takaku K., Oshima M. and Taketo M. M. (1999). Intestinal polyposis in mice with a dominant stable mutation of the β-catenin gene. *EMBO J.* 18, 5931-5942. 10.1093/emboj/18.21.5931 10545105 PMC1171659

[DMM046706C9] Hermann M., Stillhard P., Wildner H., Seruggia D., Kapp V., Sánchez-Iranzo H., Mercader N., Montoliu L., Zeilhofer H. U. and Pelczar P. (2014). Binary recombinase systems for high-resolution conditional mutagenesis. *Nucleic Acids Res.* 42, 3894-3907. 10.1093/nar/gkt1361 24413561 PMC3973285

[DMM046706C10] Ishibashi S., Brown M. S., Goldstein J. L., Gerard R. D., Hammer R. E. and Herz J. (1993). Hypercholesterolemia in low density lipoprotein receptor knockout mice and its reversal by adenovirus-mediated gene delivery. *J. Clin. Invest.* 92, 883-893. 10.1172/JCI116663 8349823 PMC294927

[DMM046706C12] Kakugawa S., Langton P. F., Zebisch M., Howell S. A., Chang T.-H., Liu Y., Feizi T., Bineva G., O'Reilly N., Snijders A. P. et al. (2015). Notum deacylates Wnt proteins to suppress signalling activity. *Nature* 519, 187-192. 10.1038/nature14259 25731175 PMC4376489

[DMM046706C13] Kellendonk C., Tronche F., Monaghan A. P., Angrand P. O., Stewart F. and Schütz G. (1996). Regulation of Cre recombinase activity by the synthetic steroid RU 486. *Nucleic Acids Res.* 24, 1404-1411. 10.1093/nar/24.8.1404 8628671 PMC145830

[DMM046706C14] Kemp R. (2004). Elimination of background recombination: somatic induction of Cre by combined transcriptional regulation and hormone binding affinity. *Nucleic Acids Res.* 32, e92-e92 10.1093/nar/gnh090 PMC443557 15247325

[DMM046706C15] Koo B.-K., Spit M., Jordens I., Low T. Y., Stange D. E., van de Wetering M., van Es J. H., Mohammed S., Heck A. J. R., Maurice M. M. et al. (2012). Tumour suppressor RNF43 is a stem-cell E3 ligase that induces endocytosis of Wnt receptors. *Nature* 488, 665-669. 10.1038/nature11308 22895187

[DMM046706C16] Kozar S., Morrissey E., Nicholson A. M., van der Heijden M., Zecchini H. I., Kemp R., Tavaré S., Vermeulen L. and Winton D. J. (2013). Continuous Clonal Labeling Reveals Small Numbers of Functional Stem Cells in Intestinal Crypts and Adenomas. *Cell Stem Cell* 13, 626-633. 10.1016/j.stem.2013.08.001 24035355

[DMM046706C17] Lopez-Garcia C., Klein A. M., Simons B. D. and Winton D. J. (2010). Intestinal stem cell replacement follows a pattern of neutral drift. *Science* 330, 822-825. 10.1126/science.1196236 20929733

[DMM046706C18] Madisen L., Garner A. R., Shimaoka D., Chuong A. S., Klapoetke N. C., Li L., van der Bourg A., Niino Y., Egolf L., Monetti C. et al. (2015). Transgenic mice for intersectional targeting of neural sensors and effectors with high specificity and performance. *Neuron* 85, 942-958. 10.1016/j.neuron.2015.02.022 25741722 PMC4365051

[DMM046706C19] Marino S., Vooijs M., van Der Gulden H., Jonkers J. and Berns A. (2000). Induction of medulloblastomas in p53-null mutant mice by somatic inactivation of Rb in the external granular layer cells of the cerebellum. *Genes Dev.* 14, 994-1004. 10783170 PMC316543

[DMM046706C20] Matsuda S., Giliberto L., Matsuda Y., McGowan E. M. and D'Adamio L. (2008). BRI2 inhibits amyloid beta-peptide precursor protein processing by interfering with the docking of secretases to the substrate. *J. Neurosci.* 28, 8668-8676. 10.1523/JNEUROSCI.2094-08.2008 18753367 PMC3844774

[DMM046706C21] Mishra S., Bernal C., Silvano M., Anand S. and Ruiz i Altaba A. (2019). The protein secretion modulator TMED9 drives CNIH4/TGFα/GLI signaling opposing TMED3-WNT-TCF to promote colon cancer metastases. *Oncogene* 38, 5817-5837. 10.1038/s41388-019-0845-z 31253868 PMC6755966

[DMM046706C22] Papafotiou G., Paraskevopoulou V., Vasilaki E., Kanaki Z., Paschalidis N. and Klinakis A. (2016). KRT14 marks a subpopulation of bladder basal cells with pivotal role in regeneration and tumorigenesis. *Nat. Commun.* 7, 11914 10.1038/ncomms11914 27320313 PMC4915139

[DMM046706C23] Park J. T. and Leach S. D. (2013). TAILOR: transgene activation and inactivation using lox and rox in zebrafish. *PLoS One* 8, e85218 10.1371/journal.pone.0085218 24391998 PMC3877360

[DMM046706C24] Plummer N. W., Evsyukova I. Y., Robertson S. D., de Marchena J., Tucker C. J. and Jensen P. (2015). Expanding the power of recombinase-based labeling to uncover cellular diversity. *Development* 142, 4385 10.1242/dev.129981 26586220 PMC4689223

[DMM046706C25] Ryder E., Doe B., Gleeson D., Houghton R., Dalvi P., Grau E., Habib B., Miklejewska E., Newman S., Sethi D. et al. (2014). Rapid conversion of EUCOMM/KOMP-CSD alleles in mouse embryos using a cell-permeable Cre recombinase. *Transgenic Res.* 23, 177-185. 10.1007/s11248-013-9764-x 24197666 PMC3890051

[DMM046706C26] Sadowski P. D. (1995). The Flp Recombinase of th 2-μm Plasmid of Saccharomyces cerevisiae. *Prog. Nucleic Acid Res. Mol. Biol.* 51, 53-91. 10.1016/S0079-6603(08)60876-4 7659779

[DMM046706C27] Sajgo S., Ghinia M. G., Shi M., Liu P., Dong L., Parmhans N., Popescu O. and Badea T. C. (2014). Dre - Cre sequential recombination provides new tools for retinal ganglion cell labeling and manipulation in mice. *PLoS One* 9, e91435 10.1371/journal.pone.0091435 24608965 PMC3946778

[DMM046706C28] Sauer B. and McDermott J. (2004). DNA recombination with a heterospecific Cre homolog identified from comparison of the pac-c1 regions of P1-related phages. *Nucleic Acids Res.* 32, 6086-6095. 10.1093/nar/gkh941 15550568 PMC534624

[DMM046706C29] Schepers A. G., Snippert H. J., Stange D. E., van den Born M., van Es J. H., van de Wetering M. and Clevers H. (2012). Lineage tracing reveals Lgr5+ stem cell activity in mouse intestinal adenomas. *Science* 337, 730-735. 10.1126/science.1224676 22855427

[DMM046706C30] Sinha M. and Lowell C. A. (2017). Efficiency and Specificity of Gene Deletion in Lung Epithelial Doxycycline-Inducible Cre Mice. *Am. J. Respir. Cell Mol. Biol.* 57, 248-257. 10.1165/rcmb.2016-0208OC 28287822 PMC5576580

[DMM046706C31] Snippert H. J., Haegebarth A., Kasper M., Jaks V., van Es J. H., Barker N., van de Wetering M., van den Born M., Begthel H., Vries R. G. et al. (2010). Lgr6 marks stem cells in the hair follicle that generate all cell lineages of the skin. *Science* 327, 1385-1389. 10.1126/science.1184733 20223988

[DMM046706C32] Sternberg N. and Hamilton D. (1981). Bacteriophage P1 site-specific recombination: I. Recombination between loxP sites. *J. Mol. Biol.* 150, 467-486. 6276557 10.1016/0022-2836(81)90375-2

[DMM046706C33] Stremmel W., Staffer S., Schneider M. J., Gan-Schreier H., Wannhoff A., Stuhrmann N., Gauss A., Wolburg H., Mahringer A., Swidsinski A. et al. (2017). Genetic Mouse Models with Intestinal-Specific Tight Junction Deletion Resemble an Ulcerative Colitis Phenotype. *J. Crohns. Colitis* 11, 1247 10.1093/ecco-jcc/jjx075 28575164 PMC5881657

[DMM046706C34] Susaki E. A. and Ueda H. R. (2016). Whole-body and Whole-Organ Clearing and Imaging Techniques with Single-Cell Resolution: Toward Organism-Level Systems Biology in Mammals. *Cell Chem. Biol.* 23, 137-157. 10.1016/j.chembiol.2015.11.009 26933741

[DMM046706C35] Van Keymeulen A., Rocha A. S., Ousset M., Beck B., Bouvencourt G., Rock J., Sharma N., Dekoninck S. and Blanpain C. (2011). Distinct stem cells contribute to mammary gland development and maintenance. *Nature* 479, 189-193. 10.1038/nature10573 21983963

[DMM046706C36] Vermeulen L., Morrissey E., van der Heijden M., Nicholson A. M., Sottoriva A., Buczacki S., Kemp R., Tavaré S. and Winton D. J. (2013). Defining stem cell dynamics in models of intestinal tumor initiation. *Science* 342, 995-998. 10.1126/science.1243148 24264992

[DMM046706C37] Vooijs M., Jonkers J. and Berns A. (2001). A highly efficient ligand-regulated Cre recombinase mouse line shows that LoxP recombination is position dependent. *EMBO Rep.* 2, 292-297. 10.1093/embo-reports/kve064 11306549 PMC1083861

[DMM046706C38] Young N. P., Crowley D. and Jacks T. (2011). Tumor and Stem Cell Biology Uncoupling Cancer Mutations Reveals Critical Timing of p53 Loss in Sarcomagenesis. *Cancer Res.* 71, 4040-4047. 10.1158/0008-5472.CAN-10-4563 21512139 PMC3160277

[DMM046706C39] Zhang Y., Proenca R., Maffei M., Barone M., Leopold L. and Friedman J. M. (1994). Positional cloning of the mouse obese gene and its human homologue. *Nature* 372, 425-432. 10.1038/372425a0 7984236

